# Increased NRG1-ErbB4 signaling in human symptomatic epilepsy

**DOI:** 10.1038/s41598-017-00207-7

**Published:** 2017-03-10

**Authors:** Jun-Ming Zhu, Ke-Xin Li, Shu-Xia Cao, Xiao-Juan Chen, Chen-Jie Shen, Ying Zhang, Hong-Yan Geng, Bi-Qing Chen, Hong Lian, Jian-Min Zhang, Xiao-Ming Li

**Affiliations:** 1 Department of Neurosurgery, Second Affiliated Hospital, Zhejiang University School of Medicine, Hangzhou, Zhejiang Province 310009 China; 2 Department of Neurobiology, Institute of Neuroscience, Key Laboratory of Medical Neurobiology of the Ministry of Health, Joint Institute for Genetics and Genome Medicine between Zhejiang University and University of Toronto, Collaborative Innovation Center for Brain Science, Zhejiang University School of Medicine, Hangzhou, Zhejiang Province 310058 China

## Abstract

Previous studies have shown that the neuregulin 1 (NRG1)-ErbB4 signaling pathway may regulate the excitability of fast-spiking neurons in the frontal cortex and participate in primary epilepsy pathogenesis. However, the exact roles and mechanism for NRG1/ErbB4 in human symptomatic epilepsy are still unclear. Using fresh human symptomatic epilepsy tissues, we found that the protein levels of NRG1 and ErbB4 were significantly increased in the temporal cortex. In addition, NRG1-ErbB4 signaling suppressed phosphorylation of GluN2B at position 1472 by Src kinase, and decreased levels of phosphorylation level of GluN2B and Src were detected in human symptomatic epilepsy tissues. Our study revealed a critical role of the NRG1-ErbB4 signaling pathway in symptomatic epilepsy, which is different from that in primary epilepsy, and we propose that the NRG1-ErbB4 signaling may act as a homeostasis modulator that protects the brain from aggravation of epileptiform activity.

## Introduction

Epilepsy is considered an intractable disease owing to the unpredictable onset and refractory nature of seizure attacks^1^. Although excessive neuronal activity is thought to be the key cause of epilepsy, the underlying mechanism is still not clear. Neuregulin 1 (NRG1) is an endogenous growth factor that regulates synaptic transmission and suppresses long-term potentiation^2–5^. ErbB4, an important NRG1 receptor, participates in many critical functions, such as neurodevelopment and synaptic plasticity^2–4^. Both NRG1 and ErbB4 have been implicated in epilepsy in mouse models and in humans^6–8^, but a precise role and mechanism for NRG1/ErbB4 in human symptomatic epilepsy, a type of epilepsy with specific etiology and organic brain disease, remain unclear.

Cavernous angioma (CA) is a congenital latent cerebrovascular disease; approximately 50% of CA patients are first identified by symptomatic epileptic discharge^9^. Here, using symptomatic epilepsy samples from CA patients, we investigated whether NRG1-ErbB4 signaling is altered in human symptomatic epilepsy and the biological consequences. We found that patients with symptomatic epilepsy showed a remarkable increase in protein levels of NRG1 and ErbB4 in the temporal cortex and a substantial decrease in the phosphorylation levels of Glu2B-pY1472 and Src-pY416, two downstream targets of NRG1-ErbB4 signaling. Our results suggest that NRG1-ErbB4 signaling may play an important role in symptomatic epilepsy and that NRG1-ErbB4 signaling may act as a homeostasis modulator that protects the brain from aggravated epileptiform activity.

## Results

Twelve CA patients with symptomatic epilepsy were informed of the study and consented to donate surgically resected tissues for research. All 12 patients were diagnosed by neuroradiologists, neurologists, and neurosurgeons, and the diagnosis was further confirmed by postoperative pathological analysis. The patients’ ages ranged from 25 to 59 years with an average age of 40 years. Disease course ranged from 2 weeks to 7 years with an average of 40 months, and the CA diameter ranged from 0.3 to 2.1 cm with an average of 1.2 cm.

**Figure 1:**
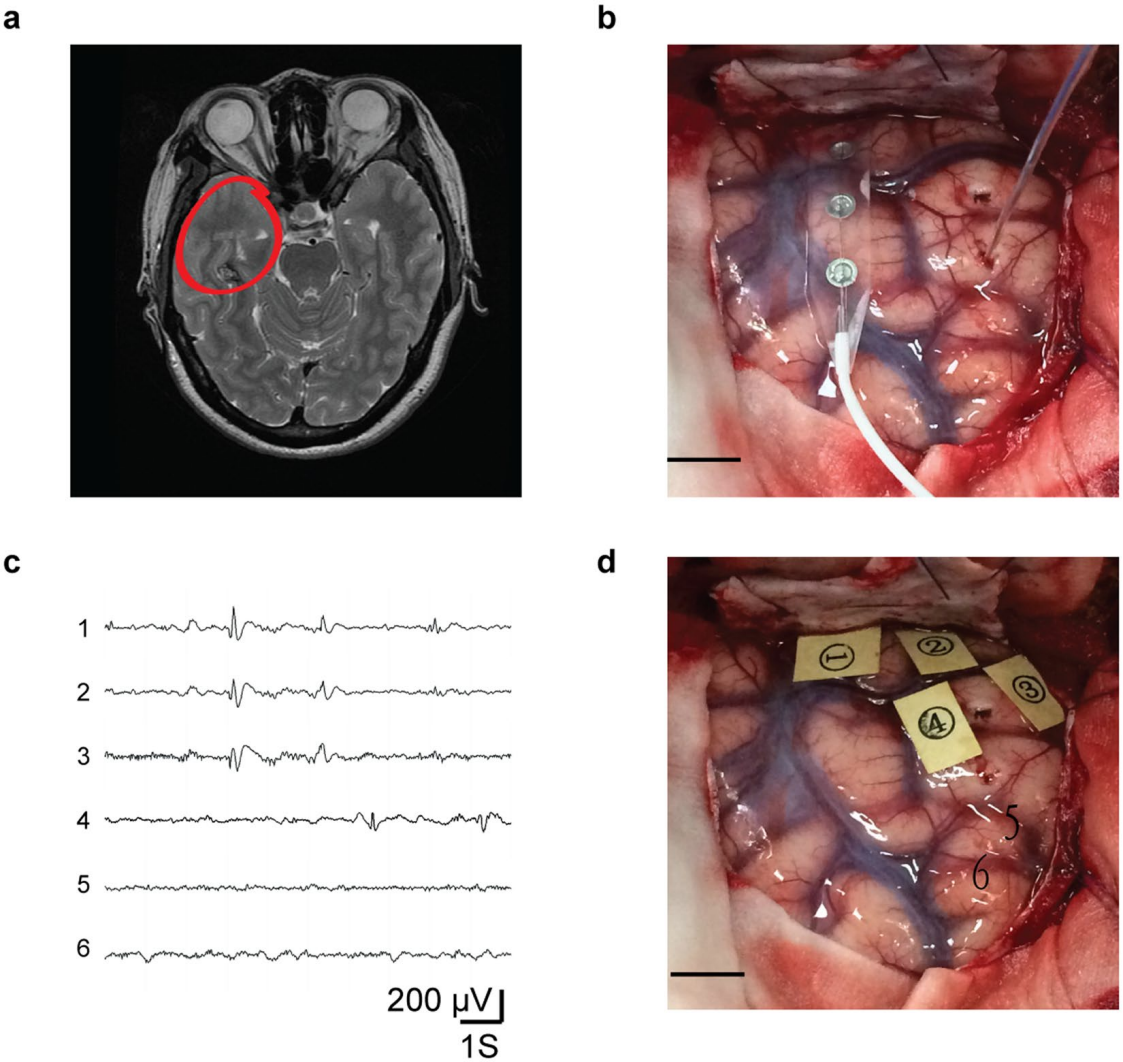
Resection of human symptomatic epileptic tissues. (**a**) Axial T2W images demonstrate a CA lesion near the right dorsal area of the hippocampus. (**b**) To confirm the resection extent of the epileptic foci, cortical electrodes and deep electrodes were used for epileptic discharge recording in the right temporal lobe during operation. (**c**) 1-4 shows the epileptic discharges recorded by cortical electrodes of four points around CA. (**d**) The area with epileptic discharges around CA were labeled with markers 1 to 4 for afterward resection.

During surgical resection, the extent of the epileptic foci was monitored by EEG recording using subdural electrodes or deep electrodes. Axial T2W images demonstrated a CA lesion near the right dorsal section of the hippocampus (Fig. 1a). EEG recording suggested that the epileptic discharge originated from the right temporal lobe (Fig. 1b). To further confirm the resection region, subdural electrodes or deep electrodes were used to monitor the epileptic discharge of each point (Fig. 1b and c), and the areas with rhythmic epileptic discharge were resected and collected for subsequent experiments (Fig. 1d). The diameter of epileptic foci ranged from 1.5 to 4.5 cm. The resected specimens used for biochemical experiments were all from the temporal lobe and CA.

### Higher expression of NRG1 and ErbB4 in human symptomatic epileptogenic tissues

**Figure 2:**
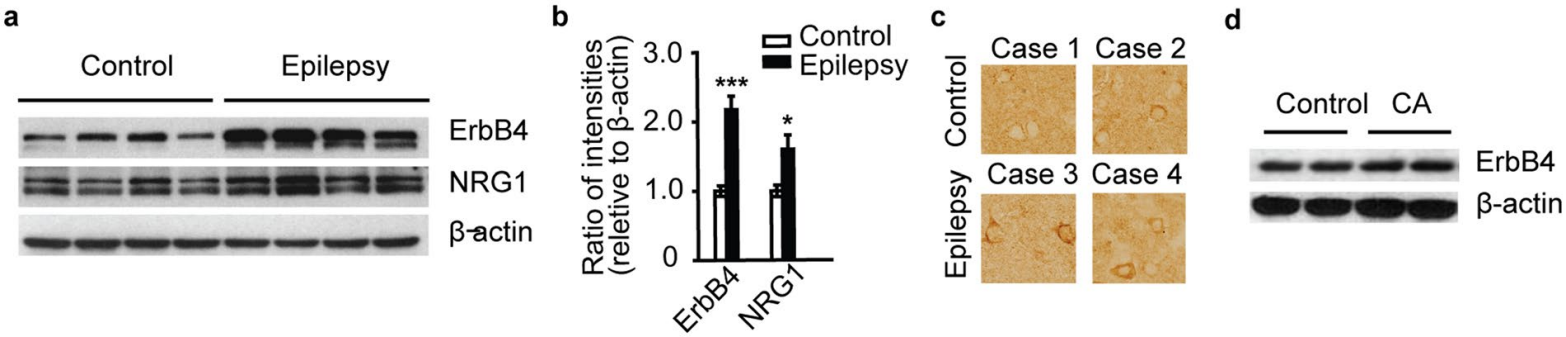
The expression of neuregulin 1 (NRG1) and ErbB4 in human symptomatic epileptic tissues. (**a**) Immunoblotting shows increased ErbB4 and NRG1 protein expression in the temporal cortex of symptomatic epilepsy patients. (**b**) Quantification of NRG1 and ErbB4 immunoblots (n = 12). (**c**) DAB staining shows increased ErbB4 immunoreactivity in symptomatic epileptic tissues. (**d**) Representative western blots of ErbB4 in cavernoma patients with and without symptomatic seizure and epileptiform discharge. Cavernoma patients without symptomatic seizure and epileptiform discharge (CA). *P < 0.05; ***P < 0.001. Unpaired two-tailed Student’s t-test. Data are represented as the mean ± s.e.m.

Previous results in mice have shown that NRG1-ErbB4 signaling regulates the activity of parvalbumin (PV)-positive neurons and that ErbB4 deletion in PV-positive neurons increases epilepsy susceptibility^6, 7^. Moreover, the ErbB4 signal is altered in temporal lobe epilepsy^6, 7^. We postulated that NRG1-ErbB4 signaling may participate in human symptomatic epilepsy and could be involved in human symptomatic epileptogenesis. Using protein lysates from human temporal lobe specimens obtained by surgical excision, we immunoblotted for NRG1 and ErbB4. Specimens from the 12 CA patients with symptomatic epilepsy were the experimental group, and temporal lobe specimens from traumatic brain injury patients without symptomatic seizure, epileptiform discharge, or other central nervous system diseases were used as the controls. The protein levels of ErbB4 and NRG1 were both drastically increased in the symptomatic epilepsy group (Fig. 2a and b). The increased ErbB4 protein was also confirmed by ErbB4 immunostaining of human symptomatic tissues (Fig. 2c).

To exclude the influence of CA, we measured the protein level of ErbB4 in specimens from CA patients without symptomatic seizure and epileptiform discharge, which were verified by EEG. There were no significant changes in ErbB4 expression in CA patients without symptomatic seizure and epileptiform discharge compared with the control group (Fig. 2d).

### GluN2B as a molecular target of NRG1-ErbB4 signaling

**Figure 3:**
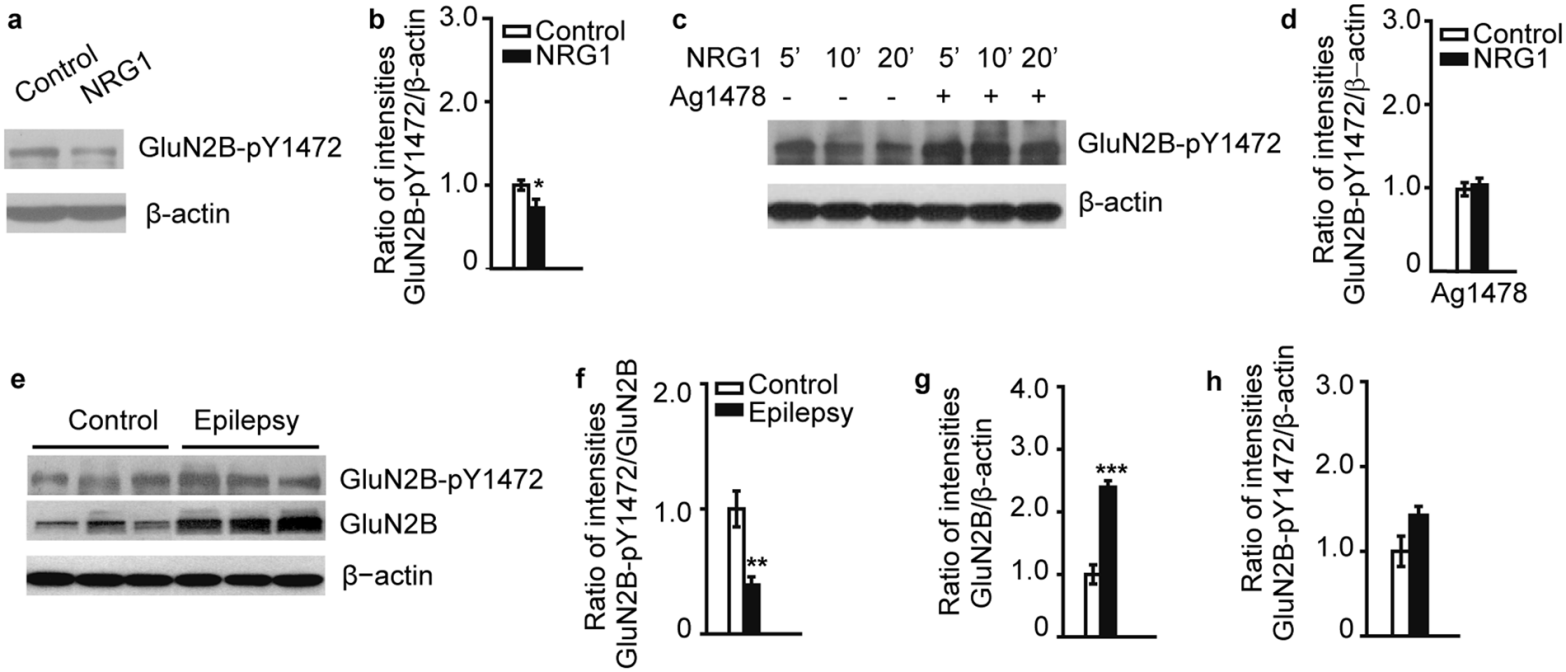
GluN2B as a downstream target of the NRG1-ErbB4 signaling in the human brain. (**a**) NRG1-treated brain slices exhibit a significant decrease in GluN2B-pY1472. (**b**) Quantification of GluN2B-pY1472 immunoblots (n = 8). (**c** and **d**) Pretreatment with the ErbB4 inhibitor Ag1478 prevented the decrease of GluN2B-pY1472 induced by NRG1 (n = 3). (**e**) Immunoblot of GluN2B-pY1472 in human symptomatic epileptic tissues. (**f**) The phosphorylation level of GluN2B at Y1472 was significantly decreased in the temporal cortex of patients with symptomatic epilepsy (n = 6 and 8). (**g**) GluN2B protein was significantly increased in symptomatic epilepsy tissues (n = 6 and 8). (**h**) The expression of GluN2B-pY1472 protein was not significantly different in symptomatic epilepsy tissues compared to control group (n = 6 and 8). *P < 0.05; **P < 0.01; ***P < 0.001. Unpaired two-tailed Student’s t-test. Data are represented as the mean ± s.e.m.

GluN2B, a component of the NMDA receptor (NMDAR), is an important downstream target of ErbB4^2, 3^. GluN2B phosphorylation is highly correlated with heightened NMDA currents that contribute to synaptic strengthening during epileptiform activity, resulting in seizure aggravation and cognitive impairment^10–17^. To test whether NRG1 regulates GluN2B phosphorylation in human tissues, human brain slices from control subjects were incubated with NRG1 and the level of GluN2B phosphorylation measured. The results showed that NRG1 incubation resulted in a suppression of GluN2B-pY1472 (Fig. 3a and b). To determine whether GluN2B-pY1472 suppression by NRG1 was dependent on ErbB4, fresh brain slices were preincubated with an ErbB4 inhibitor, Ag1478, before NRG1 incubation. The results showed that Ag1478 markedly attenuated GluN2B-pY1472 suppression induced by NRG1 (Fig. 3c and d). This result confirmed that the phosphorylation of GluN2B at Y1472 is subject to regulation by NRG1-ErbB4 signaling.

To test whether the phosphorylation level of GluN2B at Y1472 is altered in human symptomatic epileptic tissues, we used temporal cortical tissues from symptomatic epilepsy CA patients and measured the levels of GluN2B and phosphorylated GluN2B. The phosphorylation level of GluN2B at Y1472 was significantly decreased in symptomatic epileptic tissues compared with that of the controls (Fig. 3e and f). Though the protein level of GluN2B was significantly increased in tissues from symptomatic epilepsy patients (Fig. 3e and g), the expression of GluN2B-pY1472 protein was not significantly different in symptomatic epilepsy tissues compared to control group (Fig. 3e and h). Taken together, these results suggest that GluN2B may function as a molecular target of NRG1-ErbB4 signaling in human symptomatic epileptic brains.

### Regulation of GluN2B by the NRG1-ErbB4-Src signaling axis and decreased Src phosphorylation in human symptomatic epileptic brains

**Figure 4:**
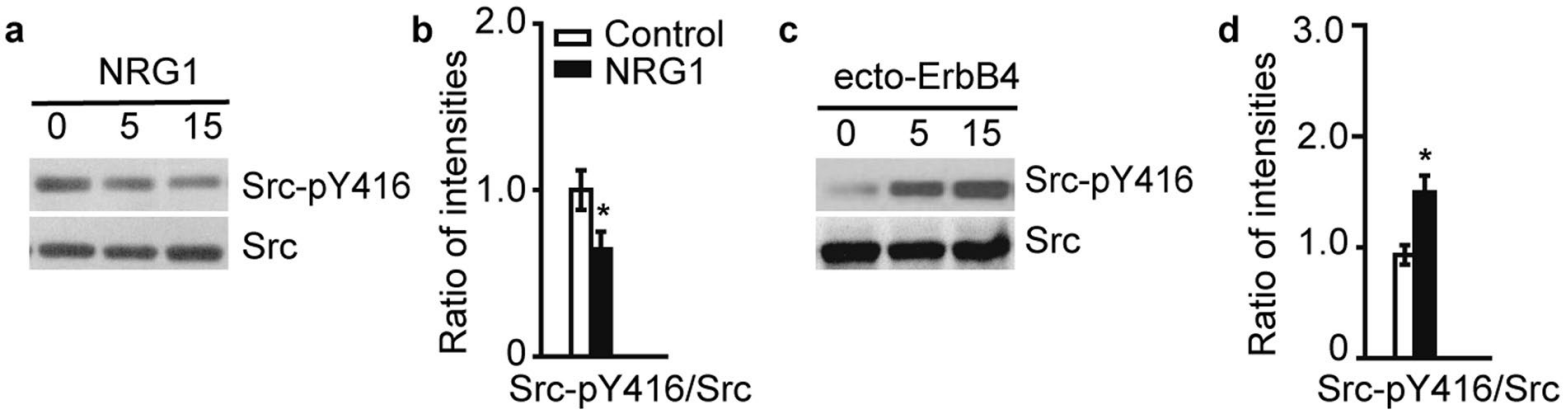
Src kinase is regulated by NRG1-ErbB4 signaling in the human brain. (**a**) NRG1 decreased the phosphorylation of Src at Y416 in the human temporal cortex. (**b**) Statistical analysis of phosphorylated Src at Y416 (n = 8). (**c**) Ecto-ErbB4 (2 μg/ml) treatment markedly increases Src-pY416 in the human brain. (**d**) Quantification of Src-pY416 immunoblots (n = 5). *P < 0.05. Unpaired two-tailed Student’s t-test. Data are represented as the mean ± s.e.m.

Previous studies have shown that Src is the predominant tyrosine kinase that leads to increased NMDAR activity and that the phosphorylation of GluN2B at Y1472 is a major target of Src^2, 3, 12, 18–20^. We tested whether the regulation of GluN2B-pY1472 by NRG1-ErbB4 is mediated by Src in human tissues. To demonstrate that Src is a downstream target of NRG1-ErbB4, acute human brain slices were incubated with NRG1 for different durations of time and Src phosphorylation was analyzed. The phosphorylation of Src at Y416 (Src-pY416), the active form of Src, was progressively suppressed by NRG1 in a time-dependent manner (Fig. 4a and b). Moreover, when we blocked endogenous NRG1 using ecto-ErbB4, an NRG1-neutralizing peptide^6^, Src phosphorylation was robustly increased (Fig. 4c and d), indicating that endogenous NRG1 is sufficient to modulate Src activity. The effect of exogenous and endogenous NRG1 on Src activation confirmed that Src is a downstream target of the NRG1-ErbB4 signaling in the human brain.

**Figure 5:**
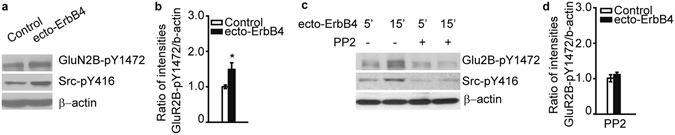
The NRG1-ErbB4 pathway regulates phosphorylation of GluN2B through Src kinase in human tissues. (**a**) Ecto-ErbB4 (2 μg/ml) treatment simultaneously increased GluN2B-pY1472 and Src-pY416 in human brain slices. (**b**) Quantification of GluN2B-pY1472 immunoblots (n = 5). (**c** and **d**) Pretreatment with the Src inhibitor PP2 prevents the increase of Glu2B-pY1472 induced by ecto-ErbB4 (n = 3). *P < 0.05. Unpaired two-tailed Student’s t-test. Data are represented as the mean ± s.e.m.

Next, we tested whether the decrease in GluN2B-pY1472 by NRG1 is mediated by Src kinase. Incubation of human brain slices with ecto-ErbB4 increased the protein levels of GluN2B-pY1472 and Src-pY416 simultaneously (Fig. 5a and b). In addition, pretreating brain slices with the Src family kinase inhibitor PP2 for 40 min fully reversed the effect of ecto-ErbB4 on GluN2B-pY1472 (Fig. 5c and d). Together, these results suggest that Src kinase plays an intermediary role between NRG-ErbB4 and GluN2B phosphorylation.

**Figure 6:**
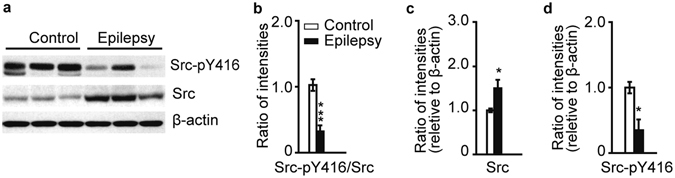
Decreased phosphorylation level of Src at Y416 in symptomatic epileptic tissues. (**a**) Immunoblotting of Src-pY416 and Src in human symptomatic epileptic tissues. (**b**) The phosphorylation of Src at Y416 is significantly decreased in human symptomatic epileptic tissues (n = 7). (**c**) Increased Src expression in human symptomatic epileptic tissues (n = 7). (**d**) The expression of Src-pY416 in human symptomatic epileptic tissues was significantly decreased (n = 7). *P < 0.05; ***P < 0.001. Unpaired two-tailed Student’s t-test. Data are represented as the mean ± s.e.m.

Using human symptomatic epileptic tissues, we observed significantly decreased phosphorylation levels of Src at 416 (Fig. 6a and b). Though the total Src protein expression was increased in symptomatic epileptic tissues compared with the control group (Fig. 6a and c), the expression of Src-pY416 in human symptomatic epileptic tissues was significantly decreased (Fig. 6a and d). These results suggest that the decreased GluN2B phosphorylation in human symptomatic epileptic tissues may be regulated by the NRG1-ErbB4-Src signaling pathway.

## Discussion

In this study, using symptomatic epilepsy tissues from CA patients, we showed that the ErbB4 was significantly increased in the brains of CA patients with symptomatic epilepsy. However, brain ErbB4 levels were similar between CA patients without symptomatic epilepsy and control groups. The difference among the three groups implies that epilepsy, but not CA, is responsible for the ErbB4 increase in CA patients with symptomatic epilepsy.

Our previous study showed that ErbB4 expression was significantly decreased in the epilepsy tissues of mesial temporal epilepsy patients^6^. In comparison, the symptomatic epilepsy tissues from the temporal lobe of CA patients presented increased ErbB4 protein levels. The discrepancy between ErbB4 expression in mesial temporal epilepsy and symptomatic epilepsy suggests that ErbB4 may play different roles in the two types of epilepsy. Previous study showed that ErbB4 deletion in PV-positive neurons increased susceptibility to epilepsy. Additionally, ErbB4 protein was significantly decreased in mesial temporal epilepsy patients, which implies that ErbB4 may be a causal factor for seizure onset in mesial temporal epilepsy. However, in symptomatic epilepsy patients, the ErbB4 signal was notably increased in the temporal lobe. Moreover, increased NRG-ErbB4 could suppress the phosphorylation of GluN2B by Src kinase. These results suggest that the increase of NRG-ErbB4 may serve as a homeostatic response of the symptomatic epilepsy brain against further aggravation induced by epileptic activity^17^. Confirmation of these or other possibilities will require more detailed investigation.

The NMDA receptor, composed of GluN1, GluN2A-2D, and GluN3A, is the major excitatory ligand-gated ion channel in the central nervous system^21^. NMDAR-dependent synaptic strengthening triggered by intractable epileptiform activity contributes to seizure aggravation and cognitive impairment. Studies have shown that the mRNA level of GluN2B instantaneously increased in a mouse epilepsy model induced by electric kindling^11, 22^. Meanwhile, it has been reported that the mRNA level of GluN2B is persistently increased in the hippocampus of a chronic epilepsy model^14, 16^. The increased GluN2B in human epilepsy tissues in this study is consistent with previous reports and implies a possible role of NMDAR in symptomatic epilepsy disease pathology^13, 14^. We confirmed that GluN2B-pY1472 is a downstream target of NRG1-ErbB4 signaling and NRG1/ErbB4 could suppress the phosphorylation of GluN2B in human tissues. Meanwhile, in human epileptic tissues, NRG1 and ErbB4 protein were significantly increased. Though the protein level of GluN2B was significantly increased in tissues from symptomatic epilepsy patients, the expression of GluN2B-pY1472 protein was not significantly different compared to control group, which suggested that the phosphorylation of GluN2B was suppressed in symptomatic epilepsy tissues. The negative correlation between NRG1-ErbB4 and the phosphorylation of GluN2B, the increased NRG1/ErbB4 and decreased phosphorylation of GluN2B at Y1472, suggests that the NRG1-ErbB4 pathway may be protective against NMDAR-mediated hyperexcitability in symptomatic epilepsy.

Src kinase inhibitors suppress the spontaneous epileptic activity of the hippocampal CA3 region in acute brain slices^19^, which suggests a role for Src kinase in epileptiform discharge. Our acute experiments in human slices demonstrated that the increase in NRG1-ErbB4 signaling can reduce Src phosphorylation. Meanwhile, we verified the decrease in Src phosphorylation in human symptomatic epilepsy tissues, though the total Src protein was increased. These findings suggest that Src is downstream of NRG1-ErbB4 signaling and that alterations of the NRG1-ErbB4-Src signaling axis may participate in symptomatic epilepsy development.

Previous studies and results from our lab in mice have showed that ErbB4 was primarily expressed in interneurons, especailly PV neurons, in prefrontal cortex, and conditional deletion of ErbB4 induced by PV-cre, but not CaMKII-cre, enhanced animal susceptibility to epilepsy^6, 7^. In addition, intracerebral infusion of NRG1 inhibited epileptogenesis, indicating that NRG1-ErbB4 signaling may be an anti-epileptogenic pathway in PV neurons in mice. In this study, using human symptomatic epileptic tissues, we found that ErbB4 protein was significantly increased in temporal cortex. While, the expression pattern of ErbB4 protein in human temporal cortex was still unclear. Which types of neurons would ErbB4 expressed and in which types of neurons ErbB4 changed, these questions were all need further study. The expression level of NRG1-ErbB4, phosphorylated GluN2B and Src kinase were all changed in human symptomatic epileptic tissues. However, whether those changes occur in a cell-type-specific manner will also require more systematic investigation.

Comorbidity is a common clinical phenomenon^23, 24^. Epidemiologic investigation showed that the probabilities of epilepsy patients developing schizophrenia or vice versa are both several-fold higher than the normal population^25–29^. However, the underlying mechanism is not clear. Combining our results with previous studies using schizophrenia patient samples^2, 3, 30^, reveals that NRG1 suppresses GluN2B tyrosine phosphorylation by Src in both schizophrenia and symptomatic epilepsy patient brain tissues. The regulation of Glu2B phosphorylation by NRG1 in both schizophrenia and symptomatic epilepsy may therefore provide some insights into the comorbidity between the two diseases.

To summarize, we found that the protein levels of NRG1 and ErbB4 were significantly increased, whereas Glu2B and Src phosphorylation were decreased in symptomatic epilepsy. The mechanism underlying these changes may be that NRG1-ErbB4 signaling suppresses GluN2B phosphorylation by Src inhibition. As an essential component of NMDAR, GluN2B is closely related to synaptic activity. The regulation of GluN2B by the NRG1-ErbB4-Src signaling axis highlighted the importance of NRG1-ErbB4 signaling in symptomatic epilepsy pathology, and the pathway we described here may offer more opportunities for anti-epileptic drug research.

## Materials and Methods

### Reagents

The NRG1 used is a recombinant polypeptide containing the entire EGF domain of β-type Neuregulin 1 (Prospec). The coding sequence for the ecto-domain of ErbB4 (amino acids 1–659, ecto-ErbB4) was subcloned into pC4DNA/Fc to generate pErbB4ex/Fc. HEK-293 cells stably expressing ecto-ErbB4 were generated and cultured in low–immunoglobulin G medium for collection of conditioned medium. ErbB4ex/Fc was purified by a HiTrap column (Amersham).

### Immunoblotting

Fresh human temporal lobe tissues were homogenized in RIPA buffer containing 50 m M Tris-HCl, pH 7.4, 150 m M NaCl, 2 m M EDTA, 1% sodium deoxycholate, 1% SDS, 1 m M PMSF, 50 m M sodium fluoride, 1 m M sodium vanadate, 1 m M DTT, and protease inhibitor cocktails. Protein lysates were loaded on 10% acrylamide SDS-PAGE gels and transferred to nitrocellulose membranes. Before incubated with primary antibodies, the membranes were incubated in TBS containing 0.1% Tween 20 and 5% milk for 1 h at room temperature (RT). Membranes were incubation with the following primary antibodies overnight at 4 °C: rabbit NRG1-specific antibody, 1:1,000, abcam; rabbit ErbB4-specific antibody, 1: 5,000, abcam; rabbit GluN2B-specific antibody, 1:1,000, Novus; rabbit GluN2B-pY1472-specific antibody, 1:1,000, Novus; rabbit Src-specific antibody, 1:2,000, Cell Signaling Technology; rabbit Src-pY416-specific antibody, 1:2,000, Cell Signaling Technology; mouse β-actin-specific antibody, 1:1,000, sigma. Next day, after three-times washing, membranes were incubated with HRP-conjugated secondary antibody in the TBS buffer for 1 h at RT. Immunoreactive bands were visualized using enhanced chemiluminescence (Pierce) and films were scanned with an Epson 1680 scanner. ImageJ (National Institutes of Health) was used for quantitative analysis.

### Slice preparation

Fresh human temporal lobe tissues were preserved in ice-cold artificial cerebrospinal fluid (ACSF) until sectioned with a Vibroslice (Leica VT 1000 S). ACSF was consisted of 125 mM NaCl, 3 mM KCl, 1.25 mM NaH_2_PO_4_, 2 mM MgSO_4_, 2 mM CaCl_2_, 25 mM NaHCO_3_ and 10 mM glucose. Slices (300 µm) were recovery for 60 min in ACSF at 33 °C before the following incubation of NRG1, ecto-ErbB4, Ag1478 (tocris) and PP2 (tocris).

### DAB Staining

Fresh human brain tissues were fixed with 4% paraformaldehyde (4 g per 100 ml saline) for 5 h and then dehydrated with 30% sucrose (15 g per 50 ml PBS) for 48 h. Brain tissues were then sectioned (40 µm) using a Vibroslice (VT 1000 S). Rinse sections with 1% H_2_O_2_ in TBS for 30 min at RT followed by 5 min rinse of sections with TBS for 3 times. Human brain sections were treated with 3% (vol/vol) normal goat serum in PBS containing 0.5% Triton X-100 for 1 h at RT. Then the brain sections were incubated with rabbit ErbB4-specific antibody (1:100, Santa Cruz) at 4 °C for 36–48 h, 10 min rinse of the human slices with TBS for 4 times. Incubate sections with vector ABC kit. Incubate at RT for 2 hours and then rinse slices with TBS 10 min for 4 times. Coloration for 5–10 mins using vector ABC kit. An upright fluorescence microscope (Olympus BX61) was used for picture capture.

### Quantification and statistical analysis

All data are presented as the mean ± s.e.m. and were analyzed using unpaired two-tailed Student’s t-test. Normality and homogeneity of variance were considered during statistic analysis. *P* < 0.05 was considered statistically significant. For quantification, values from three independent experiments with at least three biological replicates were used. The investigator was blinded to the group allocation and when assessing its outcome during experiments.

### Approval

all experimental protocols were approved by the licensing committee of Second Affiliated Hospital, Zhejiang University School of Medicine.

### Accordance

the methods were carried out in accordance with the relevant guidelines and regulations of Second Affiliated Hospital, Zhejiang University School of Medicine.

### Informed consent

Informed consent was obtained from all subjects.

## References

[1] Fisher RS (2005). Epileptic seizures and epilepsy: definitions proposed by the International League Against Epilepsy (ILAE) and the International Bureau for Epilepsy (IBE). Epilepsia.

[2] Pitcher GM (2011). Schizophrenia susceptibility pathway neuregulin 1-ErbB4 suppresses Src upregulation of NMDA receptors. Nat Med.

[3] Hahn CG (2006). Altered neuregulin 1-erbB4 signaling contributes to NMDA receptor hypofunction in schizophrenia. Nature medicine.

[4] Mei L, Xiong WC (2008). Neuregulin 1 in neural development, synaptic plasticity and schizophrenia. Nat Rev Neurosci.

[5] Law AJ, Shannon Weickert C, Hyde TM, Kleinman JE, Harrison PJ (2004). Neuregulin-1 (NRG-1) mRNA and protein in the adult human brain. Neuroscience.

[6] Li KX (2012). Neuregulin 1 regulates excitability of fast-spiking neurons through Kv1.1 and acts in epilepsy. Nat Neurosci.

[7] Tan GH (2012). Neuregulin 1 represses limbic epileptogenesis through ErbB4 in parvalbumin-expressing interneurons. Nat Neurosci.

[8] Eilam R, Pinkas-Kramarski R, Ratzkin BJ, Segal M, Yarden Y (1998). Activity-dependent regulation of Neu differentiation factor/neuregulin expression in rat brain. Proceedings of the National Academy of Sciences of the United States of America.

[9] Robinson JR, Awad IA, Little JR (1991). Natural history of the cavernous angioma. Journal of neurosurgery.

[10] Yamada N, Bilkey DK (1991). Kindling-induced persistent alterations in the membrane and synaptic properties of CA1 pyramidal neurons. Brain research.

[11] Palizvan MR, Fathollahi Y, Semnanian S, Hajezadeh S, Mirnajafizadh J (2001). Differential effects of pentylenetetrazol-kindling on long-term potentiation of population excitatory postsynaptic potentials and population spikes in the CA1 region of rat hippocampus. Brain research.

[12] Wang YT, Salter MW (1994). Regulation of NMDA receptors by tyrosine kinases and phosphatases. Nature.

[13] Mikuni N (1999). NMDA-receptors 1 and 2A/B coassembly increased in human epileptic focal cortical dysplasia. Epilepsia.

[14] Mathern GW (1997). Human hippocampal AMPA and NMDA mRNA levels in temporal lobe epilepsy patients. Brain: a journal of neurology.

[15] Kemp JA, McKernan RM (2002). NMDA receptor pathways as drug targets. Nature neuroscience.

[16] Moussa RC, Ikeda-Douglas CJ, Thakur V, Milgram NW, Gurd JW (2001). Seizure activity results in increased tyrosine phosphorylation of the N-methyl-D-aspartate receptor in the hippocampus. Brain research. Molecular brain research.

[17] Nateri AS (2007). ERK activation causes epilepsy by stimulating NMDA receptor activity. The EMBO journal.

[18] Wang, Y. T. & Salter, M. W. Regulation of NMDA receptors by tyrosine kinases and phosphatases. (1994). 10.1038/369233a0 7514272

[19] Sanna PP (2000). A role for Src kinase in spontaneous epileptiform activity in the CA3 region of the hippocampus. Proceedings of the National Academy of Sciences of the United States of America.

[20] Salter MW, Kalia LV (2004). Src kinases: a hub for NMDA receptor regulation. Nature reviews. Neuroscience.

[21] Kemp JA, McKernan RM (2002). NMDA receptor pathways as drug targets. Nature neuroscience.

[22] Al-Ghoul WM, Meeker RB, Greenwood RS (1997). Amygdala kindling alters N-methyl-D-aspartate receptor subunit messenger RNA expression in the rat supraoptic nucleus. Neuroscience.

[23] Marrie RA (2016). Sex differences in comorbidity at diagnosis of multiple sclerosis: A population-based study. Neurology.

[24] Hansen BH, Alfstad KA, van Roy B, Henning O, Lossius MI (2016). Sleep problems in children and adolescents with epilepsy: Associations with psychiatric comorbidity. Epilepsy & behavior: E&B.

[25] Altinoz AE, Tosun Meric O, Tosun Altinoz S, Essizoglu A, Cosar B (2016). Psychiatric disorders comorbid with epilepsy in a prison sample. Seizure.

[26] Landais A, Crespel A, Moulis JL, Coubes P, Gelisse P (2016). Psychiatric comorbidity in temporal DNET and improvement after surgery. Neuro-Chirurgie.

[27] Coppola A (2016). Psychiatric comorbidities in patients from seven families with autosomal dominant cortical tremor, myoclonus, and epilepsy. Epilepsy & behavior: E&B.

[28] Clancy MJ, Clarke MC, Connor DJ, Cannon M, Cotter DR (2014). The prevalence of psychosis in epilepsy; a systematic review and meta-analysis. BMC psychiatry.

[29] Bakken IJ (2014). Substance use disorders and psychotic disorders in epilepsy: a population-based registry study. Epilepsy research.

[30] Hu ML (2016). A Review of the Functional and Anatomical Default Mode Network in Schizophrenia. Neuroscience bulletin.

